# Early Prediction and Prognostic Value of Chronic Critical Illness and Persistent Inflammation, Immunosuppression, and Catabolism Syndrome in Patients with Severe Acute Pancreatitis: A Retrospective Cohort Study

**DOI:** 10.3390/jcm15083038

**Published:** 2026-04-16

**Authors:** Xuetao Zhang, Ming Chen, Beiyuan Zhang, Tao Gao, Ying Xu, Xiancheng Chen, Wenkui Yu

**Affiliations:** Department of Critical Care Medicine, Nanjing Drum Tower Hospital, Affiliated Hospital of Medical School, Nanjing University, Nanjing 210008, China; marshall1004@163.com (X.Z.); chenliuer2015@163.com (M.C.); beiyuanzhang@smail.nju.edu.cn (B.Z.); taog1983@aliyun.com (T.G.); xuying.0110@163.com (Y.X.)

**Keywords:** chronic critical illness, persistent inflammation, immunosuppression, catabolism syndrome, acute pancreatitis, severity, mortality, cohort study

## Abstract

**Background**: Patients with severe acute pancreatitis (SAP) are at high risk for developing chronic critical illness (CCI) and persistent inflammation, immunosuppression, and catabolism syndrome (PICS). However, the specific risk factors and prognostic implications of these conditions remain unclear. **Methods**: This single-center retrospective cohort study enrolled adult patients with SAP requiring ICU stay > 14 days. Least absolute shrinkage and selection operator (LASSO) regression was used to select risk factors, followed by multivariable logistic regression to identify independent predictors for CCI and PICS. Discriminative ability of early severity scores was assessed using the area under the curve (AUC) and compared via the DeLong’s test. Kaplan–Meier and Cox regression evaluated in-hospital and 90-day mortality differences. **Results**: Among 156 SAP patients, 60 (38.5%) developed CCI, 38 (24.4%) progressed to PICS, and 32 (20.5%) met both criteria. After multivariable adjustment and sensitivity analyses, the computed tomography severity index (CTSI) emerged as an independent risk factor for both CCI (odds ratio [OR]: 1.40, 95% confidence interval [CI] 1.18–1.66) and PICS (OR: 1.56, 95% CI 1.29–1.89), with AUC values of 0.747 and 0.804, respectively. Significant differences in both the in-hospital and 90-day mortality rates were observed among the three groups (*p* < 0.001). The patients meeting both CCI and PICS criteria exhibited markedly increased risks of in-hospital (hazard ratio [HR]: 7.80, 95% CI 1.53–39.77; *p* = 0.013) and 90-day mortality (HR: 3.11, 95% CI 1.08–8.95; *p* = 0.035) after the multivariable adjustment. **Conclusions**: CTSI is an independent risk factor for CCI and PICS in patients with SAP. Both CCI and PICS were associated with a heightened risk of adverse outcomes. Future studies are warranted to explore the clinical characteristics and targeted interventions for this high-risk subgroup.

## 1. Introduction

Advancements in the concepts and technologies of critical care medicine have enabled a growing number of patients to survive critical illnesses, such as sepsis, severe trauma, and severe acute pancreatitis [1,2,3]. However, a substantial proportion of hospitalized patients remain dependent on intensive care unit (ICU) support beyond the acute phase and exhibit prolonged mechanical ventilation and recurrent infections, a condition defined as chronic critical illness (CCI) [4]. A large-scale epidemiological study involving more than 3 million ICU admissions in the United States reported a CCI prevalence of approximately 34.4 per 100,000 people, with an increasing trend associated with advancing age. The in-hospital mortality rate exceeded 30%, and the one-year mortality rate surpassed 50%, with associated healthcare costs reaching approximately 26 billion dollars [5]. Moreover, survivors of CCI often experience long-term physical impairment and psychological disorders following hospital discharge, hindering their return to normal life and work, and thereby substantially escalating societal healthcare and economic burden [6,7,8]. In 2012, the Sepsis and Critical Illness Research Center at the University of Florida proposed a novel CCI endotype: persistent inflammation, immunosuppression, and catabolism syndrome (PICS) [9]. PICS may represent a potential pathophysiological paradigm for CCI and serve as its predominant clinical endotype, with age, comorbidity burden, and disease severity being identified as major risk factors [10,11]. Given the theoretical reversibility inherent to its definition, patients with PICS have been the focus of research as target populations for prevention and intervention [12].

Acute pancreatitis (AP) is a common internal medicine disease worldwide. An initial sterile inflammatory response may precipitate organ failure (OF), leading to divergent clinical trajectories [13]. According to the Revised Atlanta Classification, AP is categorized based on the presence and duration of organ failure into mild (no OF), moderately severe (OF ≤ 48 h), and severe (OF > 48 h) [14]. Severe acute pancreatitis (SAP) has a mortality rate of up to 40% and necessitates ICU admission for close monitoring and organ support [15]. SAP is characterized by an intense systemic inflammatory response followed by a variable degree of immunosuppression and catabolism, mirroring the pathophysiological trajectory of PICS. Moreover, the onset and persistence of organ failure, infected pancreatic necrosis (IPN), and other complications render patients with SAP highly susceptible to both CCI and PICS. However, this association has scarcely been investigated and early predictive markers remain elusive.

To address this gap, we conducted a retrospective cohort study at a large tertiary referral center to investigate the early clinical risk factors for the development of CCI and/or PICS in patients with SAP and characterize the subsequent adverse outcomes in this population. We found that the computed tomography severity index (CTSI) was the most useful predictor common to both CCI and PICS. Notably, patients who met the criteria for both CCI and PICS had an exceptionally poor prognosis. These findings provide a simple, clinically applicable tool for early risk stratification in SAP and highlight the compounded burden of concurrent CCI and PICS.

## 2. Materials and Methods

### 2.1. Design, Setting, and Participants

A consecutive cohort of adult patients with SAP requiring an ICU stay exceeding 14 days was retrospectively analyzed at a large tertiary teaching hospital between December 2018 and December 2024. The exclusion criteria were as follows: age < 18 years, pregnancy, time from disease onset > 7 days, incomplete medical records, and immunosuppression prior to admission. This study was approved by the Ethics Committee of Nanjing Drum Tower Hospital, Affiliated Hospital of Medical School, Nanjing University (Ethics Approval Number: 2026-0147). Due to the retrospective design of the study, the requirement for informed consent was waived by the ethics committee. This study was conducted in accordance with the principles of the Declaration of Helsinki, and all patient data were anonymized to protect privacy. The reporting of the study findings adhered to the STROBE statement [16].

### 2.2. Definitions and Outcomes

CCI was defined as an ICU length of stay ≥ 14 days, accompanied by a Day 14 Sequential Organ Failure Assessment (SOFA) score ≥ 2 (or a cardiovascular component score ≥ 1) [17]. PICS was defined by the following criteria: ICU length of stay > 14 days; C-reactive protein (CRP) > 0.5 mg/L; lymphocyte count < 0.8 × 10^9^/L; and either serum albumin < 30 g/L, body mass index (BMI) < 18 kg/m^2^, or body weight loss > 10% [18]. Data for the PICS diagnostic criteria were collected on Day 14 after ICU admission. In the absence of Day 14 data, the closest available data between Day 11 and Day 17 were utilized [19]. The diagnosis of AP required the fulfillment of at least two of the following three criteria: (1) acute upper abdominal pain, (2) serum lipase or amylase levels exceeding three times the upper limit of normal, and (3) abdominal imaging findings consistent with acute pancreatitis. Organ failure was defined according to the modified Marshall scoring system, with a score ≥ 2 in at least one of the respiratory, cardiovascular, or renal systems. SAP was defined according to the 2012 Atlanta classification as AP accompanied by persistent organ failure (>48 h) [14]. Immunosuppression was defined as the long-term use of glucocorticoids or immunosuppressive agents or the receipt of radiotherapy/chemotherapy within 30 days prior to disease onset [17]. Secondary infection referred to a newly occurring nosocomial infection identified more than 48 h after hospital admission that necessitates the initiation of new antimicrobial therapy [20]. Multidrug-resistant organisms (MDROs) were defined as bacteria that are resistant to at least one agent in three or more antimicrobial classes [21]. Infected pancreatic necrosis (IPN) was defined by the presence of either (1) gas bubbles within (peri)pancreatic necrosis on CT, accompanied by clinically relevant signs of infection, or (2) a positive culture of (peri)pancreatic necrosis obtained during the first drainage and/or necrosectomy [14]. Ninety-day mortality was defined as all-cause mortality within 90 days after Day 14 of ICU admission. Survival data after hospital discharge were obtained through monthly telephone follow-ups or routine outpatient clinic visits.

Based on the presence of CCI and/or PICS, the cohort was categorized into three groups: Group 1, non-CCI and non-PICS (NCCI + NPICS); Group 2, either CCI or PICS alone (CCI/PICS); and Group 3, both CCI and PICS (CCI + PICS). The primary outcome of this study was the discriminative performance of the early predictors of CCI or PICS development in patients with SAP. Secondary outcomes included in-hospital mortality, 90-day mortality, length of ICU stay, length of hospital stay, and hospitalization costs.

### 2.3. Data Collection

Structured medical records were retrospectively reviewed to collect the following clinical parameters: demographic characteristics (sex, age, and BMI), comorbidity burden (Charlson Comorbidity Index), laboratory findings (e.g., white blood cell count, CRP, and serum creatinine), and clinical severity scores (Acute Physiology and Chronic Health Evaluation [APACHE] II score, SOFA score, Bedside Index of Severity in Acute Pancreatitis [BISAP] score, modified Marshall score [MMS], CT severity index [CTSI], and modified CTSI [MCTSI]). The CTSI and MCTSI were assessed based on the first contrast-enhanced CT (CECT) performed after at least 72 h of symptom onset, which was obtained approximately one week after symptom onset at the discretion of the attending clinician. In contrast, all the other parameters were recorded at the time of admission. The CTSI and MCTSI were assessed by a senior expert in gastrointestinal imaging (T.G., >20 years of experience). A random subset of cases was independently reviewed by another gastrointestinal imaging expert (W.Y., >30 years of experience), and no systematic discrepancies were identified. Missing data were minimal, accounting for less than 5% of the values for the majority of the variables in this cohort (Supplementary Table S1). Consequently, an imputation using the mean or median was performed based on the distribution of the respective parameters.

### 2.4. Statistical Analysis

Due to the lack of prior references for the primary endpoint of this study, a formal sample size calculation was not performed. Instead, all the eligible patients were enrolled to maximize the statistical power of discriminative analyses. The biostatistical protocol was reviewed and approved by the Medical Statistics Center of the Nanjing Drum Tower Hospital.

Continuous variables are presented as mean ± standard deviation (SD) or as median with interquartile range (25th–75th percentiles) [M(P25–P75)], depending on the distributional characteristics. Comparisons between multiple groups were conducted using the Kruskal–Wallis rank-sum test. Categorical variables were compared using the chi-square test or Fisher’s exact test, as appropriate, based on the expected frequencies. Adjustments for multiple comparisons were performed using the Bonferroni correction.

Binary logistic regression models were constructed to identify the early risk factors for the development of CCI or PICS as separate outcomes. The variables with a *p*-value < 0.05 in univariable analysis were then screened using least absolute shrinkage and selection operator (LASSO) regression with 10-fold cross-validation, followed by multivariable logistic regression analysis to identify independent risk factors for CCI and PICS. Multicollinearity among all the candidate predictors was assessed using the variance inflation factor (VIF) prior to multivariable regression. Receiver operating characteristic curves were generated based on early severity scores, and the area under the curve (AUC) was calculated to evaluate the discriminative ability of predicting CCI and PICS. The LASSO-selected prediction model was internally validated using bootstrap methods with 500 resamples. The Youden index was used to determine the optimal cut-off values, and the DeLong test was used to compare the differences between the ROC curves.

Kaplan–Meier curves were constructed to assess the differences in in-hospital and 90-day mortality among the three groups, with comparisons made using the log-rank test. Cox proportional hazards regression models were developed to evaluate the contribution of concomitant CCI and PICS (Group 3) to mortality outcomes, with adjustments performed using hierarchical models. The predictive value of early severity scores for mortality was assessed using the AUC. Subsequently, CCI and PICS were incorporated as exposure factors into the baseline severity scores to evaluate their prognostic value for later-phase mortality prediction.

Given that the variability in the current PICS diagnostic criteria primarily pertains to the CRP threshold, a more stringent cutoff value (>20 mg/L) was applied to define PICS in the sensitivity analyses, in accordance with previous investigations (20 mg/L, 30 mg/L and 50 mg/L) [22,23,24]. Patients with a Day 14 CRP ≤ 20 mg/L were accordingly excluded. This cutoff value was selected to retain a sufficient number of positive cases in the cohort and to ensure adequate statistical power and clinical interpretability. Statistical significance was defined as a two-sided *p*-value of <0.05. All the statistical analyses were performed using SPSS Statistics (version 26.0, IBM Corp., Armonk, NY, USA) and R (version 4.5.2, R Project for Statistical Computing, Vienna, Austria).

## 3. Results

### 3.1. Baseline and Clinical Characteristics of the Study Participants

**Figure 1:**
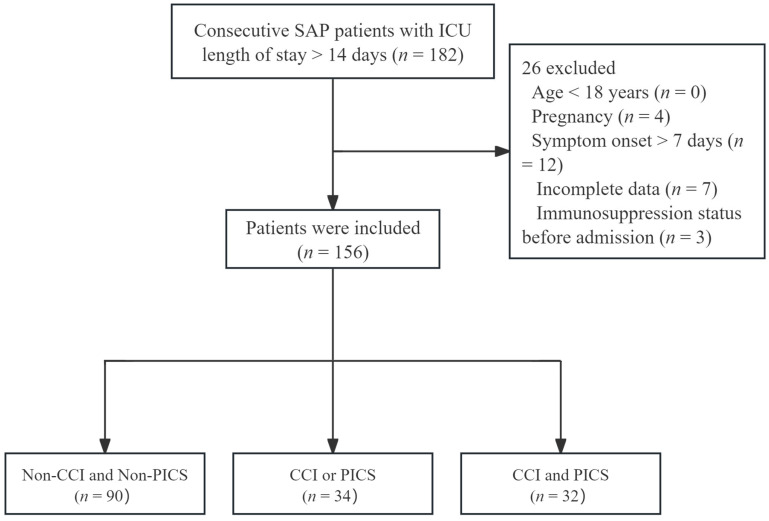
The research flow diagram of patients. SAP: severe acute pancreatitis, CCI: chronic critical illness, PICS: persistent inflammation, immunosuppression, and catabolism syndrome.

**Figure 2:**
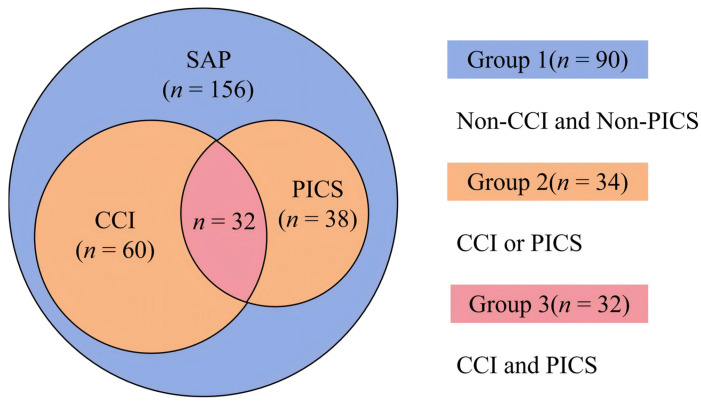
The diagram of group distribution in patients with severe acute pancreatitis. SAP: severe acute pancreatitis; CCI: chronic critical illness; PICS: persistent inflammation, immunosuppression, and catabolism syndrome.

A flowchart of the study population is shown in Figure 1. After the screening, 156 patients were included in the final analysis. Among them, 60 patients (38.5%) developed CCI and 38 patients (24.4%) progressed to PICS. Based on the presence of CCI and/or PICS, the cohort was categorized into three groups: NCCI + NPICS (*n* = 90, 57.7%), CCI/PICS (*n* = 34, 21.8%), and CCI + PICS (*n* = 32, 20.5%). The distribution of patients across these groups is shown in Figure 2. The baseline clinical characteristics are shown in Table 1. The median age of the overall cohort was 45.00 years (IQR: 35.00–59.75), and 52.60% (*n* = 82) were male. BMI was comparable across the three groups, with a mean of 26.32 ± 3.31 kg/m^2^ for the entire cohort. Baseline Charlson Comorbidity Index scores showed no significant differences among the groups, with a median of 1.00 (IQR: 0.00–2.00).

**Figure 3:**
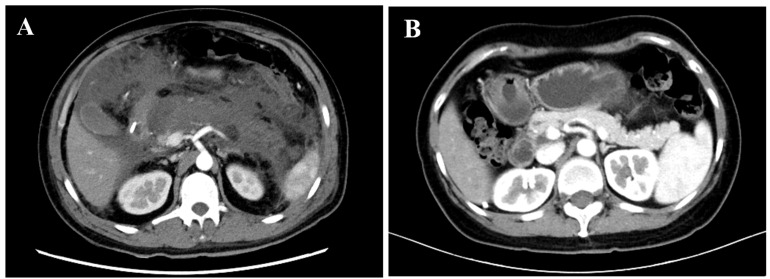
The CTSI scoring diagram for patients with SAP and a normal pancreas: (**A**) A 32-year-old male presented with persistent upper abdominal pain for 1 day and was diagnosed with severe acute biliary pancreatitis. The initial contrast-enhanced CT (obtained 7 days after symptom onset) shows extensive pancreatic necrosis with multiple peripancreatic fluid collections, yielding a CTSI score of 10. (**B**) Contrast-enhanced CT of a 51-year-old female demonstrates a normal pancreatic morphology and no peripancreatic fluid collection, with a CTSI score of 0. SAP: severe acute pancreatitis.

With the exception of MCTSI, all the early severity scores (APACHE II, SOFA, BISAP, MMS, and CTSI) differed significantly across the three groups (all *p* < 0.01), with an increasing trend corresponding to the presence of CCI and PICS, particularly for MMS and CTSI. The CTSI diagram is shown in Figure 3. Among the admission laboratory parameters, the platelet count and serum albumin levels demonstrated statistically significant differences across the groups (both *p* < 0.05). Furthermore, serum creatinine, blood urea nitrogen, and aspartate aminotransferase levels were highest in the CCI + PICS group, with significant intergroup differences (all *p* < 0.01).

### 3.2. Clinical Outcomes Across the Three Groups

Table 2 presents the clinical outcomes across the three groups, with statistically significant differences observed for all the parameters (all *p* < 0.001). The presence of CCI and/or PICS was associated with increased rates of secondary infections and infected pancreatic necrosis during the later disease course. These factors also necessitated prolonged organ support, leading to extended ICU and hospital stays, and consequently, increased overall healthcare costs. The CCI + PICS group exhibited the highest in-hospital mortality rate, with 11 deaths (34.4%), followed by the CCI/PICS group (2 deaths, 5.9%). By contrast, no in-hospital deaths occurred in the NCCI + NPICS group. The 90-day mortality rate was 43.8% (14/32) in the CCI + PICS group and 17.6% (6/34) in the CCI/PICS group. No deaths occurred within 90 days in the NCCI + NPICS group.

### 3.3. Early Predictors of CCI and PICS and Their Diagnostic Performance in Patients with SAP

The variables with *p* < 0.05 in univariable analysis were further screened using LASSO regression with 10-fold cross-validation (Supplementary Table S2 and Figure S1). All the VIF values for candidate predictors of CCI and PICS were <5 (Supplementary Table S3), indicating a low likelihood of multicollinearity. After the multivariate adjustment, as shown in Table 3, APACHE II score (OR: 1.19, 95% CI 1.05–1.36) and CTSI (OR: 1.40, 95% CI 1.18–1.66) emerged as independent risk factors for CCI. For PICS, the independent risk factors included CTSI (OR: 1.56, 95% CI 1.29–1.89) and platelet count (OR: 1.01, 95% CI 1.00–1.02).

**Figure 4:**
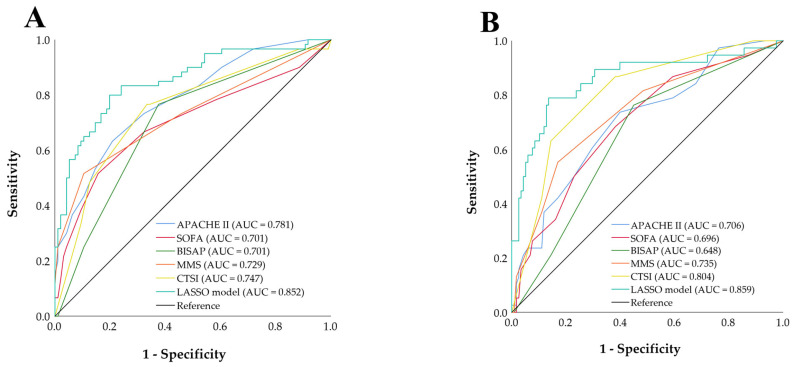
ROC curves for severity scores in predicting CCI (**A**) and PICS (**B**) in SAP patients. Composite parameters were generated by LASSO regression with 10-fold cross-validation. SAP: severe acute pancreatitis; CCI: chronic critical illness; PICS: persistent inflammation, immunosuppression, and catabolism syndrome; APACHE: Acute Physiology and Chronic Health Evaluation; SOFA: Sequential Organ Failure Assessment; BISAP: Bedside Index of Severity in Acute Pancreatitis; MMS: modified Marshall score; CTSI: computed tomography severity index; LASSO: least absolute shrinkage and selection operator.

The combined predictive probabilities derived from the logistic regression models were compared with the individual early severity scores, as illustrated in Figure 4. The LASSO-selected model achieved an AUC of 0.852 (95% CI 0.788–0.916) for predicting CCI and 0.859 (95% CI 0.780–0.937) for predicting PICS. Bootstrap validation yielded similar results (Supplementary Table S4).

Notably, CTSI alone demonstrated fair discriminative ability for both CCI and PICS, with AUC values of 0.747 (95% CI 0.666–0.828) and 0.804 (95% CI 0.736–0.881), respectively. The optimal cutoff values for CTSI were 5.5 for predicting CCI and 7.0 for predicting PICS (Supplementary Table S4). The DeLong test revealed that the AUCs of the LASSO-selected predictive models were significantly higher than those of the individual early severity scores for both CCI and PICS (all *p* < 0.05; Supplementary Table S5).

### 3.4. Kaplan–Meier Curves of In-Hospital and 90-Day Mortality Across the Three Groups

**Figure 5:**
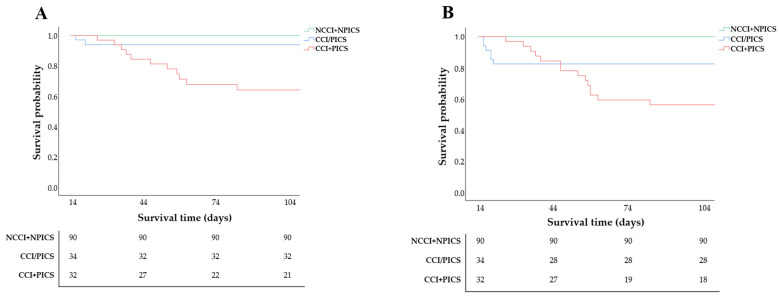
Kaplan–Meier survival curves for SAP patients among three groups: (**A**) in-hospital all-cause mortality [log-rank *p* < 0.001]; (**B**) 90-day all-cause mortality [log-rank *p* < 0.001]. SAP: severe acute pancreatitis; CCI: chronic critical illness; PICS: persistent inflammation, immunosuppression, and catabolism syndrome.

The Kaplan–Meier survival analysis revealed significant differences among the three groups for both in-hospital and 90-day mortality (log-rank *p* < 0.001 for both; Figure 5). The Cox proportional hazards regression analysis was performed to evaluate the impact of concomitant CCI and PICS on mortality outcomes. After sequential multivariable adjustment (Table 4), the coexistence of CCI and PICS remained independently associated with increased mortality risk, with a fully adjusted hazard ratio of 7.80 (95% CI 1.53–39.77; *p* = 0.013) for in-hospital mortality and 3.11 (95% CI 1.08–8.95; *p* = 0.035) for 90-day mortality.

### 3.5. ROC Curves of Early Severity Scores Combined with the CCI/PICS for Predicting In-Hospital and 90-Day Mortality

**Figure 6:**
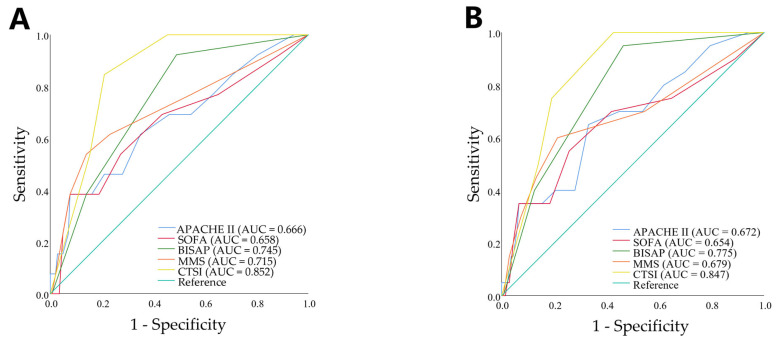
ROC curves of severity scores for predicting in-hospital (**A**) and 90-day (**B**) mortality in SAP. SAP: severe acute pancreatitis. APACHE: Acute Physiology and Chronic Health Evaluation; SOFA: Sequential Organ Failure Assessment; BISAP: Bedside Index of Severity in Acute Pancreatitis; MMS: modified Marshall score; CTSI: computed tomography severity index.

The ROC curves were generated based on early severity scores to predict in-hospital and 90-day mortality. We also assessed the later-phase prognostic value of incorporating CCI and/or PICS. As illustrated in Figure 6, CTSI demonstrated the highest discriminative ability for both in-hospital mortality (AUC = 0.852) and 90-day mortality (AUC = 0.847), followed by the BISAP score (in-hospital mortality AUC = 0.745; 90-day mortality AUC = 0.775). Furthermore, the addition of CCI and PICS significantly improved discrimination for post-Day 14 mortality (Table 5).

### 3.6. Sensitivity Analysis

After applying a more stringent CRP cutoff value and excluding patients with Day 14 CRP ≤ 20 mg/L, the revised cohort distribution is presented in Supplementary Figure S2. In the sensitivity analysis, the number of patients in the PICS (37 vs. 38) and CCI (56 vs. 60) subgroups remained almost unchanged, whereas that in the non-CCI + non-PICS group decreased substantially (55 vs. 90). Within this revised cohort, CTSI persisted as an early independent risk factor for both CCI (OR: 1.34, 95% CI 1.11–1.62) and PICS (OR: 1.56, 95% CI 1.26–1.93) (Supplementary Table S6). Furthermore, the impact of CCI and PICS on mortality outcomes remained consistent with that of the primary analysis (Supplementary Tables S7 and S8).

## 4. Discussion

In this retrospective cohort study, we found that approximately 40% of patients with SAP developed CCI and approximately 25% progressed to PICS. The early CTSI emerged as a common independent risk factor for both CCI (OR: 1.40; 95% CI 1.18–1.66) and PICS (OR: 1.56; 95% CI 1.29–1.89). The occurrence of CCI and/or PICS was associated not only with a prolonged duration of organ support and extended hospital length of stay, but also with significantly increased risks of in-hospital and 90-day mortality. Moreover, the emergence of CCI and PICS demonstrated prognostic value for later-phase mortality compared with early severity scores, including APACHE II, SOFA, BISAP, MMS, and CTSI.

This study has several strengths. First, to our knowledge, it is the first study to simultaneously evaluate the predictive value of early severity scores for both CCI and PICS in a homogeneous cohort of patients with severe acute pancreatitis. The CTSI score emerged as a clinically practical tool to predict CCI and PICS, thereby guiding early risk stratification and targeted intervention. Such patients may benefit from early nutritional optimization, intensified immunologic monitoring, and proactive management of catabolism. Second, we used LASSO regression with 10-fold cross-validation for variable selection and performed bootstrap internal validation to assess model optimism, thereby reducing the risk of overfitting and providing more robust estimates of predictive performance. Furthermore, the robustness of our primary findings is supported, to some extent, by subgroup analyses that yielded consistent inferences (Supplementary Figure S3). Third, we employed a landmark analysis with time zero set at Day 14, which eliminates guarantee-time bias and ensures that the associations between CCI/PICS and the post-Day 14 outcomes are unbiased. The prognostic value of Day 14 CCI/PICS status beyond admission scores supports the use of these phenotypes for risk stratification in the subacute phase, potentially guiding decisions regarding tracheostomy timing, nutritional support, and disposition planning. Finally, we performed sensitivity analyses using higher CRP thresholds to address the variability in the PICS diagnostic criteria, which strengthens the generalizability of our conclusions.

In addition to CTSI, the APACHE II score demonstrated good predictive performance and correlation with CCI/PICS and mortality outcomes in our analysis. As one of the most commonly used severity scores in the ICU setting, APACHE II has been identified as an important predictor of mortality in severe pancreatitis, as well as a predictor of PICS in patients with severe trauma and a reliable discriminator of mortality in patients with CCI following brain injury [25,26,27]. Although both APACHE II and CTSI are widely used in clinical practice, their relatively complex assessment components limit their applicability in certain scenarios. Specifically, the CTSI requires contrast-enhanced CT imaging, which poses considerable challenges for patients with renal impairment or in resource-limited settings. Future predictive models incorporating readily available, real-time clinical parameters may help address this clinical challenge. Interestingly, patients meeting criteria for both CCI and PICS had significantly higher platelet counts compared with other groups. While thrombocytopenia is commonly observed in sepsis and critical illness, reactive thrombocytosis can occur in the setting of persistent inflammation and ongoing tissue repair. Elevated platelet counts may also reflect activation of the acute-phase response and release of pro-inflammatory mediators. This finding underscores the complex hematologic adaptations in patients with different critical illness trajectories and warrants further investigation.

Both the CTSI and MCTSI are pancreatitis-specific severity scoring systems. Compared to the CTSI, the MCTSI simplifies the grading of pancreatic necrosis while incorporating an assessment of extrapancreatic complications [28,29]. Previous studies evaluating the utility of various severity scores in predicting AP severity and adverse outcomes have reported inconsistent results. However, the CTSI has consistently demonstrated favorable predictive performance [30,31], which aligns with the findings of the present study. In contrast to previous investigations, our study cohort comprised patients with SAP requiring ICU admission, all of whom experienced at least one episode of persistent organ failure. Even in this critically ill population, the CTSI maintained robust predictive value for the development of post-SAP chronic critical illness and adverse outcomes. While MCTSI is often regarded as more comprehensive due to its inclusion of extrapancreatic complications, CTSI’s emphasis on pancreatic necrosis and peripancreatic inflammation—both key drivers of prolonged organ failure and catabolism in SAP—may explain its superior prognostic performance in the specific setting of long-stay ICU patients. This observation suggests that, relative to extrapancreatic complications or other severity scoring systems, the extent of pancreatic necrosis may represent a critical determinant in the progression to chronic critical status in patients with SAP, potentially by increasing the risk of infected pancreatic necrosis [32].

The delayed recovery of critically ill patients and the associated adverse outcomes have been widely recognized. The concept of the CCI was introduced approximately four decades ago. Although the underlying concepts have evolved, the diagnostic criteria remain unclear. In contrast, the concept of PICS originated in the surgical intensive care unit in 2012 and identified a clinical phenotype characterized by persistent inflammation, immunosuppression, and catabolism based on the CCI. Subsequent studies have elaborated on this concept [33,34]. Although the clinical diagnostic criteria for PICS have not been fully established, their diagnostic framework has gained broad acceptance. Both concepts reflect the researchers’ recognition of and concern about the inability of patients in the later stages of critical illness to achieve rapid recovery. However, few clinical studies have discussed these factors simultaneously in a single disease cohort. This study found that the vast majority of patients with PICS (32/38, 84.2%) also met the CCI criteria for patients with SAP. This aligns with the current understanding of the relationship between the two. PICS may represent the most prominent endotype of CCI and provide a paradigm for its pathophysiological mechanisms. Nevertheless, the substantial overlap cannot exclude the possibility of misclassifications arising from the uncertainty in the diagnostic criteria. The two syndromes share a common clinical feature—prolonged ICU length of stay—which may represent the shared adverse outcome of the two sides of the same coin: persistent organ failure (namely CCI) and persistent inflammation–immunosuppression–catabolism status (PICS). Notably, individuals meeting only the CCI or PICS criteria (*n* = 34, 21.8%) also experienced a prolonged duration of organ failure, extended hospital stay, and increased mortality risk, suggesting that other endotypes warrant further exploration. Therefore, future studies should clarify the concepts and diagnostic criteria for CCI and PICS.

This study has some limitations. First, the single-center retrospective design and relatively small sample size limit the generalizability of our findings and reduce statistical power. Future large-scale, multicenter studies are needed to validate our conclusions. Second, this study only enrolled patients with an ICU length of stay exceeding 14 days, thereby automatically excluding those who died early or recovered rapidly, which may introduce selection and survival bias. Consequently, our findings apply only to patients who survived the early phase and may not generalize to the broader SAP population. However, our study primarily focuses on the development and progression of CCI/PICS and their potential adverse outcomes. Therefore, in accordance with the definitional framework of these conditions, we restricted inclusion to patients who had survived at least 14 days in the ICU. This criterion ensured that all enrolled patients had passed the early acute phase and were at risk for developing CCI and PICS. Furthermore, we conducted a landmark analysis with Day 14 of ICU stay as time zero for survival analysis, thereby enhancing the robustness and reliability of our findings. Third, the lack of formal interobserver agreement represents a potential limitation, as it may affect the validity of the CTSI scores and consequently the accuracy of our conclusions. Future studies should include formal Kappa statistics to further confirm interobserver reliability. Fourth, the number of outcome events—particularly for in-hospital mortality (*n* = 13)—was small, and despite LASSO selection and bootstrap validation, the risk of overfitting remains. Last, the lack of established diagnostic criteria for CCI and PICS may introduce potential confounding bias and limit the translational applicability of our conclusions. Nevertheless, we adopted the most commonly used CCI criteria in the literature to minimize the potential adverse effects [11]. Furthermore, we employed a more stringent CRP threshold in the sensitivity analysis, which enhanced the robustness of our findings.

## 5. Conclusions

This study comprehensively investigated the risk factors for developing CCI and PICS in patients with SAP and their adverse prognostic implications. Early severity scores, combined with laboratory parameters, effectively predicted the occurrence of CCI and PICS. Notably, the CTSI alone demonstrated favorable predictive performance for CCI, PICS, and mortality outcomes, outperforming other severity scoring systems and suggesting broad clinical applicability. The development of the CCI and/or PICS further increased the risk of adverse outcomes in patients with SAP and added later-course prognostic information to early severity scores. However, given the single-center, retrospective design and the selection bias inherent to the prolonged-ICU inclusion criterion, these findings should be regarded as hypothesis-generating. Prospective, multicenter studies are warranted to validate the predictive utility of CTSI in broader SAP populations and to further clarify the prognostic implications of combined CCI and PICS.

## Figures and Tables

**Table 1:** Baseline clinical characteristics among three groups.

Characteristics	All (*n* = 156)	NCCI + NPICS (*n* = 90)	CCI/PICS (*n* = 34)	CCI + PICS (*n* = 32)	*p*
Age (years)	45.00(35.00, 59.75)	44.00(35.00, 58.00)	53.00(39.00, 67.00)	45.00(35.00, 60.00)	0.270
Male Sex, *n* (%)	82(52.60)	45(50.00)	22(64.70)	15(46.90)	0.131
BMI (kg/m^2^)	26.32 ± 3.31	26.31 ± 3.38	26.50 ± 3.60	26.16 ± 2.83	0.920
Charlson Comorbidity Index (score)	1.00(0.00, 2.00)	1.00(0.00, 2.00)	1.00(0.00, 3.00)	1.00(0.00, 2.00)	0.059
APACHE II (score)	12.00(9.00, 15.00)	11.00(8.00, 13.00) ^a,b^	14.00(11.00, 18.00)	15.00(13.00, 19.50)	**<0.001**
SOFA (score)	4.00(3.00, 6.00)	4.00(3.00, 5.00) ^b^	5.00(3.00, 7.00)	6.00(4.50, 8.00)	**<0.001**
BISAP (score)	3.00(2.00, 3.00)	2.00(2.00, 3.00) ^a,b^	3.00(2.00, 3.00)	3.00(3.00, 3.50)	**<0.001**
MMS (score)	3.00(2.00, 4.00)	2.00(2.00, 3.00) ^a,b^	3.00(2.00, 4.00) ^c^	4.00(3.00, 6.00)	**<0.001**
CTSI (score)	5.50(4.00, 8.00)	4.00(4.00, 6.00) ^a,b^	6.00(4.00, 8.00) ^c^	8.00(6.00, 10.00)	**<0.001**
MCTSI (score)	7.00(7.00, 9.00)	7.00(7.00, 9.00)	7.00(7.00, 8.00)	7.00(7.00, 9.00)	0.497
WBC (10^9^/L)	14.95(11.43, 18.40)	14.95(11.10, 18.40)	15.10(13.00, 19.20)	14.10(11.00, 18.20)	0.669
LYM (10^9^/L)	0.90(0.70, 1.20)	1.00(0.70, 1.20)	0.80(0.60, 1.20)	0.80(0.55, 1.25)	0.484
HGB (g/L)	138.21 ± 32.80	140.02 ± 32.39	139.41 ± 30.77	131.81 ± 36.14	0.466
PLT (10^9^/L)	178.91 ± 61.99	177.73 ± 59.49	159.26 ± 59.46 ^c^	203.09 ± 65.25	**0.015**
CRP (mg/L)	220.13 ± 73.87	214.80 ± 71.71	217.11 ± 80.13	238.33 ± 72.49	0.293
BUN (mmol/L)	6.95(4.40, 10.33)	5.65(4.01, 8.20) ^a,b^	7.88(5.10, 14.30)	9.30(6.85, 14.95)	**<0.001**
Cr (µmol/L)	68.00(52.00, 119.63)	62.75(49.00, 85.00) ^b^	83.45(48.20, 186.00)	154.0(61.35, 246.35)	**<0.001**
ALT (U/L)	28.65(17.05, 68.55)	26.50(16.60, 45.40)	27.05(17.20, 69.40)	35.95(21.10, 170.75)	0.134
AST (U/L)	36.65(26.00, 76.18)	32.65(24.00, 56.00) ^b^	35.50(29.40, 74.60)	64.80(31.15, 105.40)	**0.004**
ALB (g/L)	33.25(30.65, 36.50)	34.25(31.40, 37.00) ^b^	32.40(29.50, 35.50)	31.90(29.85, 34.20)	**0.011**

CCI: chronic critical illness; PICS: persistent inflammation, immunosuppression, and catabolism syndrome; BMI: body mass index; APACHE: Acute Physiology and Chronic Health Evaluation; SOFA: Sequential Organ Failure Assessment; BISAP: Bedside Index of Severity in Acute Pancreatitis; MMS: modified Marshall score; CTSI: computed tomography severity index; MCTSI: modified CTSI; WBC: white blood cell; LYM: lymphocyte; HGB: hemoglobin; PLT: platelet; CRP: C-reactive protein; BUN: blood urea nitrogen; Cr: creatinine; ALT: alanine aminotransferase; AST: aspartate aminotransferase; ALB: albumin. Bold values indicate significant difference among three groups (*p* < 0.05). ^a^: NCCI + NPICS vs. CCI/PICS, *p* < 0.05; ^b^: NCCI + NPICS vs. CCI + PICS, *p* < 0.05; ^c^: CCI/PICS vs. CCI + PICS, *p* < 0.05.

**Table 2:** Comparison of clinical outcomes among three groups.

Characteristics	NCCI + NPICS (*n* = 90)	CCI/PICS (*n* = 34)	CCI + PICS (*n* = 32)	*p*
ICU LOS (days)	18.00(16.00, 22.00) ^a,b^	22.00(18.00, 32.00) ^c^	47.00(30.00, 60.00)	**<0.001**
Hospital LOS (days)	29.00(22.00, 37.00) ^a,b^	56.50(22.00, 99.00) ^c^	72.50(48.00, 115.50)	**<0.001**
Hospital care costs (thousand CNY)	78.98(56.03, 119.82) ^a,b^	210.06(132.89, 308.85) ^c^	542.18(359.35, 787.84)	**<0.001**
Secondary infection, *n* (%)	21.00(23.30) ^a,b^	22.00(64.70) ^c^	30.00(93.80)	**<0.001**
MDRO, *n* (%)	15.00(16.70) ^a,b^	16.00(47.10) ^c^	27.00(84.40)	**<0.001**
IPN, *n* (%)	12.00(13.30) ^a,b^	20.00(58.80) ^c^	29.00(90.60)	**<0.001**
OF duration (days)	6.50(4.00, 10.00) ^a,b^	15.00(9.00, 19.00) ^c^	36.50(25.50, 50.00)	**<0.001**
MV duration (days)	0.00(0.00, 0.00) ^a,b^	0.00(0.00, 3.00)	0.50(0.00, 14.50)	**<0.001**
Vasopressors duration (days)	0.00(0.00, 0.00) ^a,b^	0.00(0.00, 2.00) ^c^	5.00(0.00, 9.50)	**<0.001**
RRT duration (days)	0.00(0.00, 0.00) ^a,b^	0.00(0.00, 4.00) ^c^	8.50(0.00, 18.00)	**<0.001**
In-hospital mortality, *n* (%)	0.00(0.00) ^b^	2.00(5.90) ^c^	11.00(34.40)	**<0.001**
90-day mortality, *n* (%)	0.00(0.00) ^a,b^	6.00(17.60)	14.00(43.80)	**<0.001**

CCI: chronic critical illness; PICS: persistent inflammation, immunosuppression, and catabolism syndrome; ICU: intensive care unit; LOS: length of stay; CNY: Chinese Yuan; MDRO: multidrug-resistant organisms; IPN: infected pancreatic necrosis; OF: organ failure; MV: mechanical ventilation; RRT: renal replacement therapy. Bold values indicate significant difference among three groups (*p* < 0.05). ^a^: NCCI + NPICS vs. CCI/PICS, *p* < 0.05; ^b^: NCCI + NPICS vs. CCI + PICS, *p* < 0.05; ^c^: CCI/PICS vs. CCI + PICS, *p* < 0.05.

**Table 3:** Univariate and multivariate logistic regression to identify risk factors for CCI and PICS.

	CCI				PICS			
	Univariable Analysis		Multivariable Analysis		Univariable Analysis		Multivariable Analysis	
	OR (95% CI)	*p*	OR (95% CI)	*p*	OR (95% CI)	*p*	OR (95% CI)	*p*
APACHE II	1.335(1.199, 1.486)	**<0.001**	1.191(1.045, 1.357)	**0.009**	1.169(1.074, 1.273)	**<0.001**		
BISAP	2.687(1.671, 4.322)	**<0.001**			1.902(1.172, 3.087)	**0.009**		
MMS	2.185(1.605, 2.973)	**<0.001**			1.172(1.327, 2.208)	**<0.001**		
CTSI	1.461(1.254, 1.702)	**<0.001**	1.399(1.176, 1.664)	**<0.001**	1.610(1.355, 1.913)	**<0.001**	1.560(1.291, 1.885)	**<0.001**
Creatinine	1.009(1.004, 1.014)	**<0.001**			-	-		
Platelets	-	-			1.007(1.001, 1.013)	**0.029**	1.011(1.003, 1.019)	**0.006**

CCI: chronic critical illness; PICS: persistent inflammation, immunosuppression, and catabolism syndrome; APACHE: Acute Physiology and Chronic Health Evaluation; BISAP: Bedside Index of Severity in Acute Pancreatitis; MMS: modified Marshall score; CTSI: computed tomography severity index. Bold values indicate significant difference between two groups (*p* < 0.05).

**Table 4:** Cox proportional hazard ratios for in-hospital and 90-day mortality.

	Model 1		Model 2		Model 3	
	HR (95% CI)	*p*	HR (95% CI)	*p*	HR (95% CI)	*p*
In-hospital all-cause mortality						
Group 1 + 2 (*n* = 124)	Reference		Reference		Reference	
Group 3 (*n* = 32)	24.84(5.49, 112.40)	**<0.001**	24.84(5.49, 112.40)	**<0.001**	7.80(1.53, 39.77)	**0.013**
90-day all-cause mortality						
Group 1 + 2 (*n* = 124)	Reference		Reference		Reference	
Group 3 (*n* = 32)	10.28(3.94, 26.83)	**<0.001**	9.68(3.69, 25.39)	**<0.001**	3.11(1.08, 8.95)	**0.035**

Group 1 (non-CCI + non-PICS), Group 2 (CCI/PICS), and Group 3 (CCI + PICS). Model 1: unadjusted; Model 2: adjusted for sex, age, and Charlson Comorbidity Index; Model 3: Model 2 + Acute Physiology and Chronic Health Evaluation II, Bedside Index of Severity in Acute Pancreatitis, and computed tomography severity index. HR: hazard ratio; CI: confidence interval. Bold values indicate significant differences between any two groups (*p* < 0.05).

**Table 5:** Diagnostic performance of early severity scores and adjusted models in predicting in-hospital and 90-day mortality.

	Model 1		Model 2		Model 3		Model 4	
	AUC	*p*	AUC	*p* ^a^	AUC	*p* ^b^	AUC	*p* ^c^
In-hospital all-cause mortality								
APACHE II	0.666	Reference	0.834	**0.003**	0.842	**0.007**	0.898	**0.001**
SOFA	0.658	Reference	0.849	**0.002**	0.837	**0.008**	0.902	**0.001**
BISAP	0.745	Reference	0.882	**<0.001**	0.894	**0.008**	0.924	**0.001**
MMS	0.715	Reference	0.865	**0.010**	0.858	**0.030**	0.908	**0.006**
CTSI	0.852	Reference	0.915	**0.001**	0.903	0.054	0.931	**0.002**
90-day all-cause mortality								
APACHE II	0.672	Reference	0.845	**<0.001**	0.809	**0.014**	0.895	**0.004**
SOFA	0.654	Reference	0.862	**<0.001**	0.778	**0.017**	0.892	**<0.001**
BISAP	0.775	Reference	0.909	**<0.001**	0.868	**0.023**	0.923	**<0.001**
MMS	0.679	Reference	0.865	**<0.001**	0.795	**0.033**	0.893	**<0.001**
CTSI	0.847	Reference	0.923	**<0.001**	0.876	0.108	0.924	**<0.001**

Model 1: unadjusted; Model 2: +CCI; Model 3: +PICS; Model 4: +CCI, PICS. *p* ^a^: Model 2 vs. Model 1; *p* ^b^: Model 3 vs. Model 1; *p* ^c^: Model 4 vs. Model 1. CCI: chronic critical illness; PICS: persistent inflammation, immunosuppression, and catabolism syndrome; APACHE: Acute Physiology and Chronic Health Evaluation; SOFA: Sequential Organ Failure Assessment; BISAP: Bedside Index of Severity in Acute Pancreatitis; MMS: modified Marshall score; CTSI: computed tomography severity index. Bold values indicate significant differences between any two models (*p* < 0.05).

## Data Availability

Access to data supporting this study can be granted by the corresponding author upon reasonable request.

## Supplementary Materials

The following supporting information can be downloaded at https://www.mdpi.com/article/10.3390/jcm15083038/s1. Figure S1. Candidate selection using LASSO regression with 10-fold cross-validation for CCI (A,B) and PICS (C,D); Figure S2. The diagram of group distribution based on an adjusted CRP cutoff of 20 mg/L in severe acute pancreatitis; Figure S3. Subgroup analysis comparing group 3 with group 1 + 2 for in-hospital (A) and 90-day (B) mortality; Table S1. Detailed baseline clinical characteristics among three groups; Table S2. Candidate predictors and their coefficients selected by LASSO regression with 10-fold cross-validation; Table S3. The VIF values of candidate predictors for CCI and PICS; Table S4. Diagnostic performance and threshold values of LASSO-selected models and early severity scores for prediction of CCI and PICS based on ROC curves; Table S5. DeLong’s test for pairwise AUC comparisons across LASSO-selected models and early severity scores in predicting CCI and PICS; Table S6. Univariate and multivariate logistic regression to identify risk factors for CCI and PICS on an adjusted CRP cutoff of 20 mg/L in severe acute pancreatitis; Table S7. Cox proportional hazard ratios for in-hospital and 90-day mortality based on an adjusted CRP cutoff of 20 mg/L in severe acute pancreatitis; Table S8. Diagnostic performance of early severity scores and adjusted models in predicting in-hospital and 90-day mortality via ROC curves on an adjusted CRP cutoff of 20 mg/L in severe acute pancreatitis.

## Abbreviations

The following abbreviations are used in this manuscript:

CCI	Chronic Critical Illness
PICS	Persistent Inflammation, Immunosuppression, and Catabolism Syndrome
SAP	Severe Acute Pancreatitis
LASSO	Least Absolute Shrinkage and Selection Operator
ICU	Intensive Care Unit
OF	Organ Failure
CRP	C-Reactive Protein
BMI	Body Mass Index
APACHE	Acute Physiology and Chronic Health Evaluation
SOFA	Sequential Organ Failure Assessment
BISAP	Bedside Index of Severity in Acute Pancreatitis
MMS	Modified Marshall Score
CTSI	Computed Tomography Severity Index
MCTSI	Modified CTSI
AUC	Area Under the Curve
OR	Odds Ratio
HR	Hazard Ratio
CI	Confidence Interval
