# Supplementary material for: Early Prediction and Prognostic Value of Chronic Critical Illness and Persistent Inflammation, Immunosuppression, and Catabolism Syndrome in Patients with Severe Acute Pancreatitis: A Retrospective Cohort Study
Source: J Clin Med. 2026 Apr 16;15(8):3038. doi: 10.3390/jcm15083038 (PMC13116712; doi:10.3390/jcm15083038)
Supplement: Supplementary file 1 [file jcm-15-03038-s001.zip › jcm-4204030-supplementary.pdf]

Supplementary Table S1 Detailed baseline clinical characteristics among three groups

Characteristics	All (n = 156)	NCCI+NPICS (n = 90)	CCI/PICS (n = 34)	CCI+PICS (n = 32)	*P*	Missing n(%)
Age (years)	45.00(35.00, 59.75)	44.00(35.00, 58.00)	53.00(39.00, 67.00)	45.00(35.00, 60.00)	0.270	0
Male Sex, n (%)	82(52.60)	45(50.00)	22(64.70)	15(46.90)	0.131	0
BMI (kg/m^2)	26.32 ± 3.31	26.31 ± 3.38	26.50 ± 3.60	26.16 ± 2.83	0.920	7(4.49)
Charlson Comorbidity Index (score)	1.00(0.00, 2.00)	1.00(0.00, 2.00)	1.00(0.00, 3.00)	1.00(0.00, 2.00)	0.059	0
APACHE II (score)	12.00(9.00, 15.00)	11.00(8.00, 13.00)[a,b]	14.00(11.00, 18.00)	15.00(13.00, 19.50)	**< 0.001**	7(4.49)
SOFA (score)	4.00(3.00, 6.00)	4.00(3.00, 5.00)[b]	5.00(3.00, 7.00)	6.00(4.50, 8.00)	**< 0.001**	3(1.92)
BISAP (score)	3.00(2.00, 3.00)	2.00(2.00, 3.00)[a,b]	3.00(2.00, 3.00)	3.00(3.00, 3.50)	**< 0.001**	5(3.21)
MMS (score)	3.00(2.00, 4.00)	2.00(2.00, 3.00)[a,b]	3.00(2.00, 4.00)[c]	4.00(3.00, 6.00)	**< 0.001**	0
CTSI (score)	5.50(4.00, 8.00)	4.00(4.00, 6.00)[a,b]	6.00(4.00, 8.00)[c]	8.00(6.00, 10.00)	**< 0.001**	0
MCTSI (score)	7.00(7.00, 9.00)	7.00(7.00, 9.00)	7.00(7.00, 8.00)	7.00(7.00, 9.00)	0.497	0
WBC (10^9/L)	14.95(11.43, 18.40)	14.95(11.10, 18.40)	15.10(13.00, 19.20)	14.10(11.00, 18.20)	0.669	5(3.21)

LYM (10^9/L)	0.90(0.70, 1.20)	1.00(0.70, 1.20)	0.80(0.60, 1.20)	0.80(0.55, 1.25)	0.484	5(3.21)
HGB (g/L)	138.21 ± 32.80	140.02 ± 32.39	139.41 ± 30.77	131.81 ± 36.14	0.466	2(1.28)
PLT (10^9/L)	178.91 ± 61.99	177.73 ± 59.49	159.26 ± 59.46[c]	203.09 ± 65.25	**0.015**	4(2.56)
CRP (mg/L)	220.13 ± 73.87	214.80 ± 71.71	217.11 ± 80.13	238.33 ± 72.49	0.293	8(5.13)
BUN (mmol/L)	6.95(4.40, 10.33)	5.65(4.01, 8.20)[a,b]	7.88(5.10, 14.30)	9.30(6.85, 14.95)	**< 0.001**	3(1.92)
Cr (μmol/L)	68.00(52.00, 119.63)	62.75(49.00, 85.00)[b]	83.45(48.20, 186.00)	154.0(61.35, 246.35)	**< 0.001**	3(1.92)
ALT (U/L)	28.65(17.05, 68.55)	26.50(16.60, 45.40)	27.05(17.20, 69.40)	35.95(21.10, 170.75)	0.134	9(5.77)
AST (U/L)	36.65(26.00, 76.18)	32.65(24.00, 56.00)[b]	35.50(29.40, 74.60)	64.80(31.15, 105.40)	**0.004**	5(3.21)
ALB (g/L)	33.25(30.65, 36.50)	34.25(31.40, 37.00)[b]	32.40(29.50, 35.50)	31.90(29.85, 34.20)	**0.011**	4(2.56)
Day 14 CRP (mg/L)	41.45(20.03, 94.18)	23.75(12.50, 45.6)[a,b]	66.15(39.20, 129.60)	104.75(68.25, 173.95)	**< 0.001**	8(5.13)
Day 14 LYM (10^9/L)	1.10(0.70, 1.40)	1.30(0.90, 1.60)[a,b]	0.75(0.60, 1.20)	0.75(0.55, 0.90)	**< 0.001**	5(3.21)
Day 14 ALB (g/L)	35.75(32.90, 39.20)	38.20(35.20, 40.60)[a,b]	33.35(31.40, 36.60)	32.25(29.95, 35.50)	**< 0.001**	4(2.56)
Day 14 SOFA (score)	0.50(0.00, 3.00)	0.00(0.00, 0.00)[a,b]	2.50(2.00, 5.00)	4.50(3.00, 7.00)	**< 0.001**	3(1.92)

First CECT from symptom onset (days)	7.00(5.00, 8.00)	7.00(5.00, 9.00)	6.00(5.00, 7.00)	7.00(6.00, 9.50)	0.244	0

CCI: chronic critical illness; PICS: persistent inflammation, immunosuppression, and catabolism syndrome; BMI: body mass index; APACHE: Acute Physiology and Chronic Health Evaluation; SOFA: Sequential Organ Failure Assessment; BISAP: Bedside Index of Severity in Acute Pancreatitis; MMS: Modified Marshall Score; CTSI: Computed Tomography Severity Index; MCTSI: Modified CTSI; WBC: White blood cell; LYM: Lymphocyte; HGB: Hemoglobin; PLT: Platelets; CRP: C-reactive protein; BUN: Blood urea nitrogen; Cr: Creatinine; ALT: Alanine aminotransferase; AST: Aspartate aminotransferase; ALB: Albumin; CECT: contrast-enhanced CT. Bold values indicate significant difference among three groups ($P < 0.05$). [a]: NCCI+NPICS vs. CCI/PICS, $P < 0.05$; [b]: NCCI+NPICS vs. CCI+PICS, $P < 0.05$; [c]: CCI/PICS vs. CCI+PICS, $P < 0.05$.

Supplementary Table S2 Candidate predictors and their coefficients selected by LASSO regression with 10-fold cross-validation

Variables	Intercept	APACHE II	BISAP	MMS	CTSI	Creatinine	Platelets
CCI	-0.5248	0.5175	0.1154	0.2800	0.4710	0.0482	-
PICS	-1.3916	0.0721	0.0334	0.4183	0.8078	-	0.2633

CCI: chronic critical illness; PICS: persistent inflammation, immunosuppression, and catabolism syndrome; APACHE: Acute Physiology and

Chronic Health Evaluation; BISAP: Bedside Index of Severity in Acute Pancreatitis; MMS: Modified Marshall Score; CTSI: Computed Tomography Severity Index.

Supplementary Table S3 The VIF values of candidate predictors for CCI and PICS

Variables	APACHE II	BISAP	MMS	CTSI	Creatinine	Platelets
CCI	1.888	1.283	2.386	1.092	1.721	-
PICS	1.875	1.295	1.813	1.104	-	1.038

CCI: chronic critical illness; PICS: persistent inflammation, immunosuppression, and catabolism syndrome; APACHE: Acute Physiology and Chronic Health Evaluation; BISAP: Bedside Index of Severity in Acute Pancreatitis; MMS: Modified Marshall Score; CTSI: Computed Tomography Severity Index.

Supplementary Table S4 Diagnostic performance and threshold values of LASSO-selected models and early severity scores for prediction of CCI and PICS based on ROC curves

Variables	AUC	95% CI		*P* value	Sensitivity	Specificity	Cut-off
CCI							
APACHE II	0.781	0.708	0.855	**< 0.001**	0.633	0.792	13.500
SOFA	0.701	0.611	0.791	**< 0.001**	0.517	0.844	5.500
BISAP	0.701	0.617	0.786	**< 0.001**	0.767	0.625	2.500
MMS	0.729	0.642	0.815	**< 0.001**	0.517	0.896	3.500
CTSI	0.747	0.666	0.828	**< 0.001**	0.767	0.667	5.500
LASSO-selected model	0.852	0.788	0.916	**< 0.001**	0.800	0.802	0.347
Bootstrap-validated AUC	0.862	0.790	0.919	-	-	-	-
PICS							
APACHE II	0.706	0.612	0.800	**< 0.001**	0.737	0.602	12.500
SOFA	0.696	0.601	0.792	**< 0.001**	0.684	0.619	4.500
BISAP	0.648	0.552	0.745	**0.006**	0.763	0.551	2.500

MMS	0.735	0.640	0.830	**< 0.001**	0.553	0.831	2.500
CTSI	0.804	0.736	0.881	**< 0.001**	0.632	0.856	7.000
LASSO-selected model	0.859	0.780	0.937	**< 0.001**	0.789	0.864	0.294
Bootstrap-validated AUC	0.853	0.765	0.927	-	-	-	-

CCI: chronic critical illness; PICS: persistent inflammation, immunosuppression, and catabolism syndrome; AUC: area under curves; CI: confidence interval; APACHE: Acute Physiology and Chronic Health Evaluation; SOFA: Sequential Organ Failure Assessment; BISAP: Bedside Index of Severity in Acute Pancreatitis; MMS: Modified Marshall Score; CTSI: Computed Tomography Severity Index. Bold values indicate significant difference between two groups ($P < 0.05$).

Supplementary Table S5 DeLong's test for pairwise AUC comparisons across LASSO-selected models and early severity scores in predicting CCI and PICS

CCI	APACHE II	SOFA	BISAP	MMS	CTSI	LASSO-selected model
APACHE II	-	0.072	0.072	0.203	0.515	**0.016**
SOFA	0.072	-	0.994	0.457	0.430	**< 0.001**

BISAP	0.072	0.994	-	0.573	0.418	**< 0.001**
MMS	0.203	0.457	0.573	-	0.733	**0.001**
CTSI	0.515	0.430	0.418	0.733	-	**0.002**
LASSO-selected model	**0.016**	**< 0.001**	**< 0.001**	**0.001**	**0.002**	-
PICS	APACHE II	SOFA	BISAP	MMS	CTSI	LASSO-selected model
APACHE II	-	0.855	0.285	0.550	0.096	**0.003**
SOFA	0.855	-	0.377	0.323	0.060	**0.002**
BISAP	0.285	0.377	-	0.088	**0.004**	**< 0.001**
MMS	0.550	0.323	0.088	-	0.201	**0.006**
CTSI	0.096	0.060	**0.004**	0.201	-	**0.038**
LASSO-selected model	**0.003**	**0.002**	**< 0.001**	**0.006**	**0.038**	-

AUC: area under curves; CCI: chronic critical illness; PICS: persistent inflammation, immunosuppression, and catabolism syndrome; APACHE: Acute Physiology and Chronic Health Evaluation; SOFA: Sequential Organ Failure Assessment; BISAP: Bedside Index of Severity in Acute Pancreatitis; MMS: Modified Marshall Score; CTSI: Computed Tomography Severity Index; LASSO: least absolute shrinkage and selection operator. Bold values indicate significant difference between two groups ($P < 0.05$).

Supplementary Table S6 Univariate and multivariate logistic regression to identify risk factors for CCI and PICS on an adjusted CRP cutoff of 20 mg/L in severe acute pancreatitis

Variables	CCI				PICS			
	Univariable analysis		Multivariable analysis		Univariable analysis		Multivariable analysis	
	OR (95% CI)	*P*	OR (95% CI)	*P*	OR (95% CI)	*P*	OR (95% CI)	*P*
APACHE II	1.312(1.164, 1.480)	**< 0.001**	1.181(1.020, 1.366)	**0.026**	1.146(1.046, 1.255)	**0.003**		
SOFA	-	-			1.244(1.062, 1.458)	**0.007**		
BISAP	2.770(1.590, 4.827)	**< 0.001**			-	-		
MMS	1.945(1.408, 2.687)	**< 0.001**			1.541(1.186, 2.001)	**0.001**		
CTSI	1.435(1.206, 1.708)	**< 0.001**	1.338(1.106, 1.619)	**0.003**	1.565(1.297, 1.889)	**< 0.001**	1.561(1.264, 1.929)	**< 0.001**
Platelets	-	-			1.008(1.001, 1.015)	**0.017**	1.014(1.005, 1.024)	**0.002**

CCI: chronic critical illness; PICS: persistent inflammation, immunosuppression, and catabolism syndrome; APACHE: Acute Physiology and Chronic Health Evaluation; SOFA: Sequential Organ Failure Assessment; BISAP: Bedside Index of Severity in Acute Pancreatitis; MMS:

Modified Marshall Score; CTSI: Computed Tomography Severity Index. Bold values indicate significant difference between two groups (*P* < 0.05).

Supplementary Table S7 Cox proportional hazard ratios for in-hospital and 90-day mortality based on an adjusted CRP cutoff of 20 mg/L in severe acute pancreatitis

	Model 1		Model 2		Model 3	
	HR (95% CI)	*P*	HR (95% CI)	*P*	HR (95% CI)	*P*
In-hospital all-cause mortality						
Gouup 1+2 (n = 86)	Reference		Reference		Reference	
Group 3 (n = 31)	17.56(3.88, 79.48)	< 0.001	17.56(3.88, 79.48)	< 0.001	6.81(1.38, 33.72)	0.019

90-day all-cause mortality						
Gouup 1+2 (n = 86)	Reference		Reference		Reference	
Group 3 (n = 31)	7.28(2.79, 18.99)	< 0.001	6.76(2.57, 17.77)	< 0.001	2.81(0.99, 7.89)	0.050

Group 1 (non-CCI+non-PICS), Group 2 (CCI/PICS), Group 3 (CCI+PICS). Model 1: unadjusted; Model 2: adjusted for sex, age, Charlson Comorbidity Index; Model 3: Model 2 + Acute Physiology and Chronic Health Evaluation II, Bedside Index of Severity in Acute Pancreatitis, Computed Tomography Severity Index. HR: hazard ratio; CI: confidence interval. Bold values indicate significant differences between any two groups ($P < 0.05$).

Supplementary Table S8 Diagnostic performance of early severity scores and adjusted models in predicting in-hospital and 90-day mortality via ROC curves on an adjusted CRP cutoff of 20 mg/L in severe acute pancreatitis

	Model 1	Model 2	Model 3	Model 4

	AUC	*P*	AUC	*P*[a]	AUC	*P*[b]	AUC	*P*[c]
In-hospital all-cause mortality								
APACHE II	0.636	Reference	0.792	**0.003**	0.812	**0.010**	0.868	**0.001**
SOFA	0.638	Reference	0.816	**0.002**	0.808	**0.012**	0.872	**0.001**
BISAP	0.733	Reference	0.852	**0.001**	0.868	**0.022**	0.902	**0.003**
MMS	0.695	Reference	0.828	**0.008**	0.829	**0.032**	0.880	**0.006**
CTSI	0.814	Reference	0.890	**0.002**	0.872	0.081	0.909	**0.001**
90-day all-cause mortality								
APACHE II	0.640	Reference	0.805	**< 0.001**	0.771	**0.028**	0.863	**0.010**
SOFA	0.635	Reference	0.832	**< 0.001**	0.748	**0.035**	0.860	**< 0.001**
BISAP	0.769	Reference	0.887	**< 0.001**	0.842	0.091	0.905	**< 0.001**
MMS	0.647	Reference	0.827	**< 0.001**	0.761	**0.044**	0.860	**< 0.001**

CTSI	0.808	Reference	0.899	**< 0.001**	0.839	0.173	0.899	**0.001**

Model 1: unadjusted; Model 2: +CCI; Model 3: +PICS; Model 4: +CCI, PICS. Pa: Model 2 VS Model 1; Pb: Model 3 VS Model 1; Pc: Model 4 VS Model 1. CCI: chronic critical illness; PICS: persistent inflammation, immunosuppression, and catabolism syndrome; APACHE: Acute Physiology and Chronic Health Evaluation; SOFA: Sequential Organ Failure Assessment; BISAP: Bedside Index of Severity in Acute Pancreatitis; MMS: Modified Marshall Score; CTSI: Computed Tomography Severity Index. Bold values indicate significant differences between any two models ($P < 0.05$).

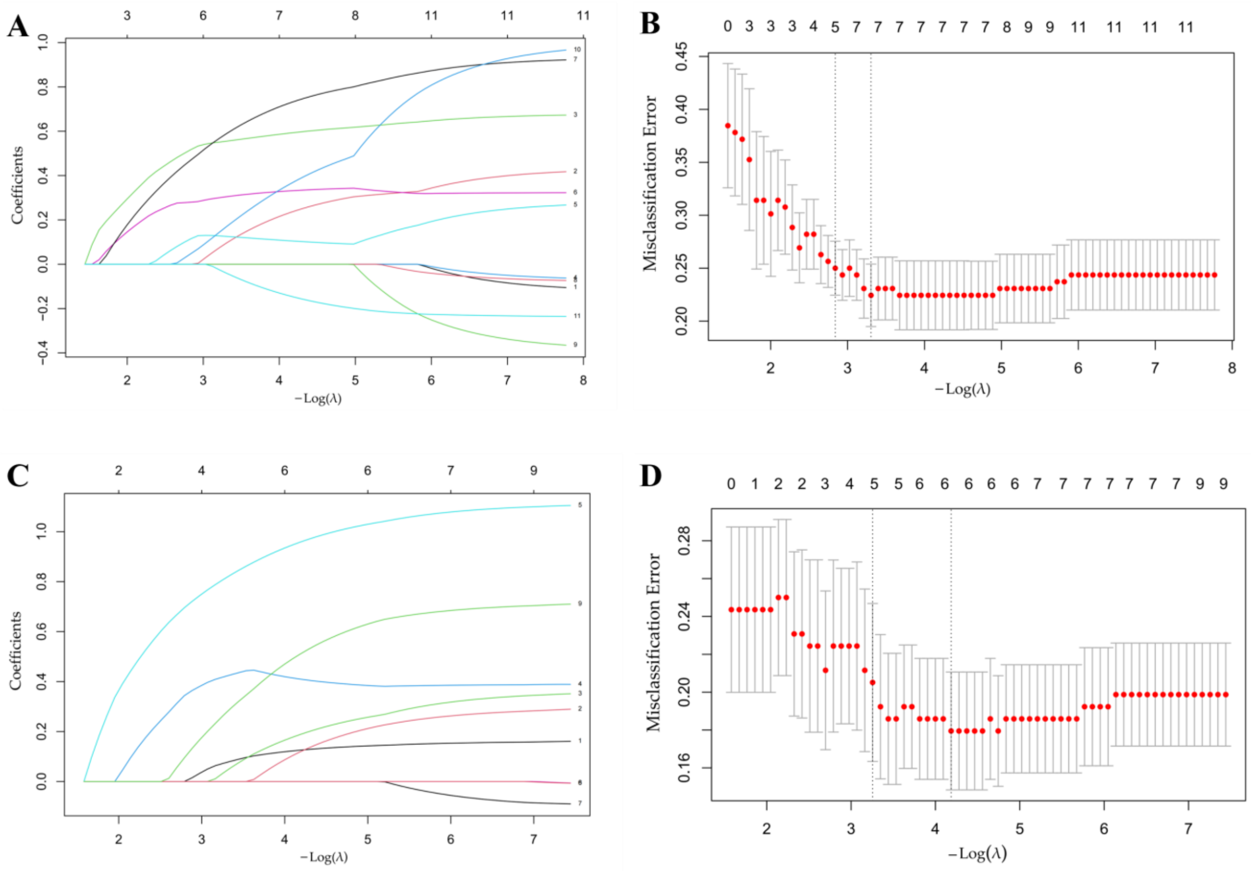

Supplementary Figure S1 Candidate selection using LASSO regression with 10-fold cross-validation for CCI (A,B) and PICS (C,D): (A) LASSO coefficient paths for CCI; (B) cross-validation error curve for CCI; (C) LASSO coefficient paths for PICS; (D) cross-validation error curve for PICS. CCI: chronic critical illness; PICS: persistent inflammation, immunosuppression, and catabolism syndrome; LASSO: least absolute shrinkage and selection operator.

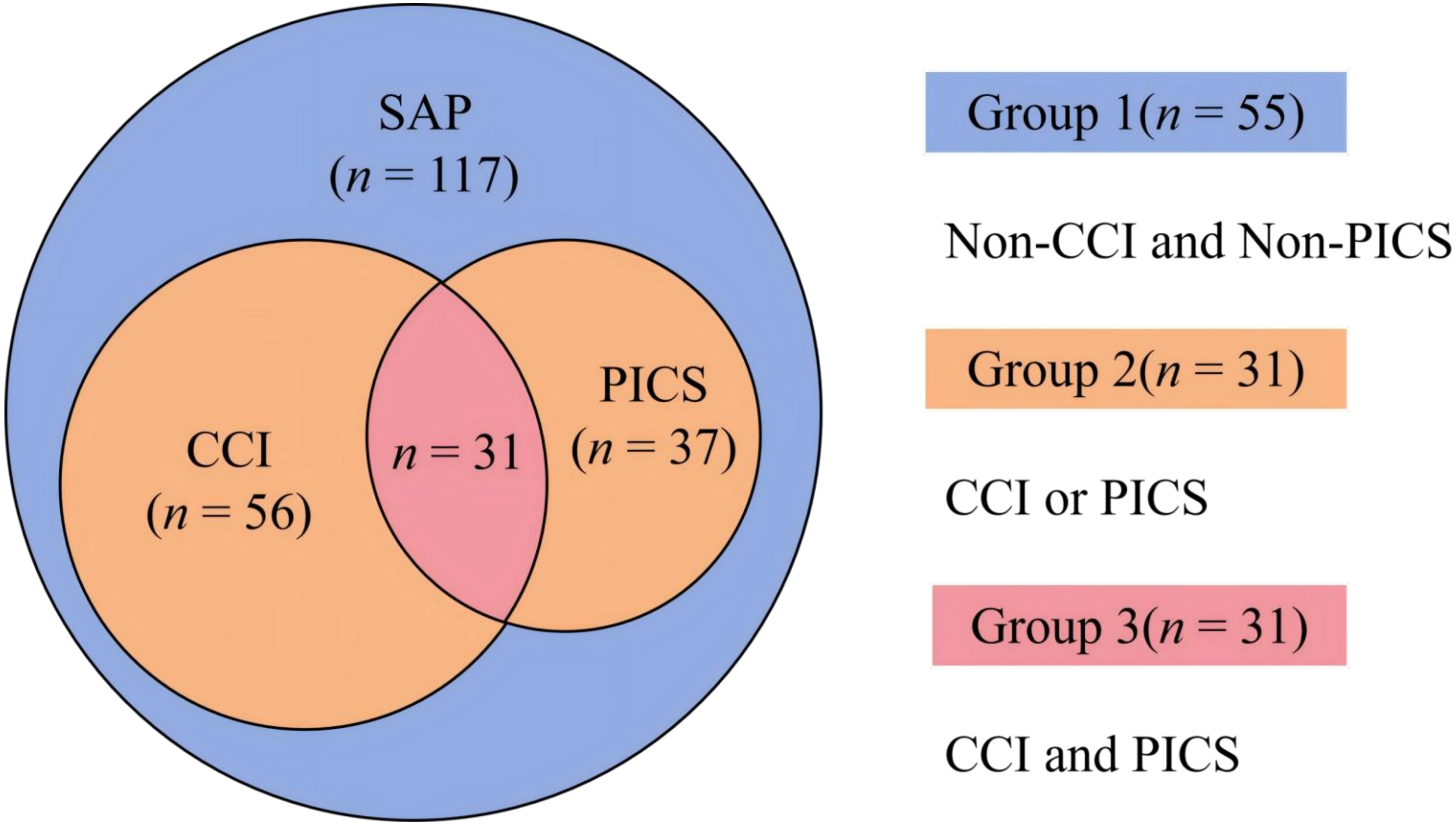

Supplementary Figure S2 The diagram of group distribution based on an adjusted CRP cutoff of 20 mg/L in severe acute pancreatitis. CCI: chronic critical illness; PICS: persistent inflammation, immunosuppression, and catabolism syndrome.

A

In-hospital Mortality: Group 3 vs Group 1+2

Subgroup	Events/Total	HR (95% CI)	P Value	P-interaction
Age ≥50	5 / 66	15.69 (1.75-140.77)	0.01	0.62
Age <50	8 / 90	33.76 (4.14-275.37)	0.001	
Gender Male	6 / 82	9.04 (1.65-49.39)	0.01	1.00
Gender Female	7 / 74	5198460869.98 (0.00-Inf)	1.00	
APACHEII ≥16	6 / 36	7.26 (0.85-62.25)	0.07	0.27
APACHEII <16	7 / 120	44.16 (5.29-368.72)	<0.001	
SOFA ≥6	7 / 46	11.17 (1.34-93.21)	0.03	0.48
SOFA <6	6 / 110	36.75 (4.28-315.62)	0.001	
MOF yes	7 / 39	6.45 (0.77-53.74)	0.08	0.20
MOF no	6 / 117	47.40 (5.51-407.82)	<0.001	

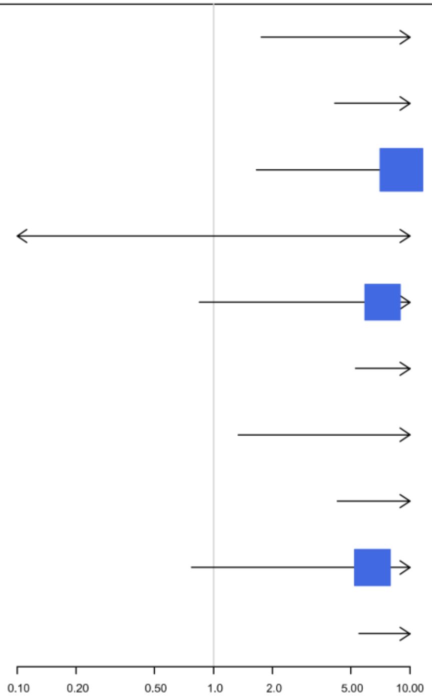

B

90-day Mortality: Group 3 vs Group 1+2

Subgroup	Events/Total	HR (95% CI)	P Value	P-interaction
Age ≥50	11 / 66	4.64 (1.41-15.25)	0.01	0.09
Age <50	9 / 90	39.16 (4.88-314.02)	<0.001	
Gender Male	10 / 82	4.56 (1.32-15.77)	0.02	0.11
Gender Female	10 / 74	39.21 (4.95-310.80)	<0.001	
APACHEII ≥16	8 / 36	4.49 (0.90-22.31)	0.07	0.28
APACHEII <16	12 / 120	13.90 (4.17-46.35)	<0.001	
SOFA ≥6	11 / 46	5.00 (1.32-18.94)	0.02	0.31
SOFA <6	9 / 110	14.08 (3.51-56.48)	<0.001	
MOF yes	11 / 39	2.89 (0.76-10.91)	0.12	0.06
MOF no	9 / 117	18.28 (4.55-73.41)	<0.001	

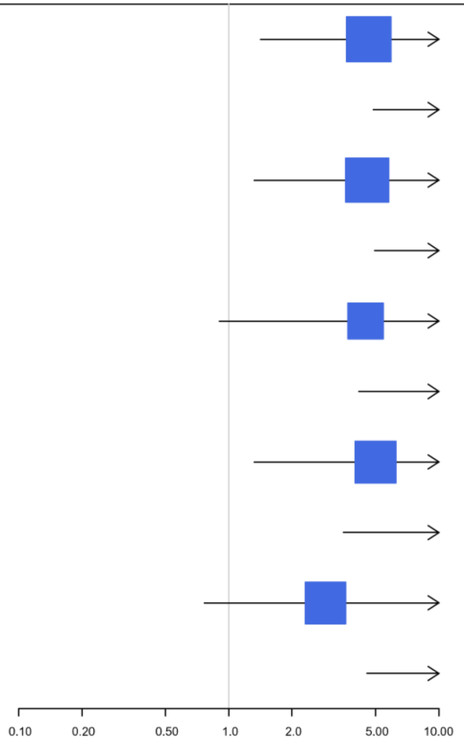

Supplementary Figure S3 Subgroup analysis comparing group 3 with group 1 + 2 for in-hospital (A) and 90-day (B) mortality. Group 1(non-CCI + non-PICS), Group 2(CCI/PICS), Group 3(CCI + PICS). CCI: chronic critical illness; PICS: persistent inflammation, immunosuppression, and catabolism syndrome; APACHE: Acute Physiology and Chronic Health Evaluation; SOFA: Sequential Organ Failure Assessment; MOF: multiple organ failure.
